# Angular momentum of a circularly polarized light beam

**DOI:** 10.1016/j.heliyon.2024.e37993

**Published:** 2024-09-19

**Authors:** R.I. Khrapko

**Affiliations:** Moscow Aviation Institute National Research University, Russia

**Keywords:** Classic spin, Electrodynamics, Spin tensor

## Abstract

It is shown that the Maxwell electrodynamics cannot explain the existence of an angular momentum flux in a circularly polarized light beam which has no the phase structure. The fact is that such a beam contains a small rotating electromagnetic mass and, accordingly, contains an angular momentum directed along the beam axis, but this rotating mass has no speed in the direction of the beam axis. Therefore, this angular momentum is not transferred along the beam axis, and there is no flow of this angular momentum along the beam axis. We show the angular momentum flux in such a beam is contained in the main mass of the beam, which does not rotate. This angular momentum flux is a spin flux and is described by the spin tensor. Likewise, taking spin into account is required to explain the classical Beth experiment and other experiments.

## 1 Introduction

Since the 19th century it has been known [1,2] that circularly polarized light contains *internal* angular momentum, in addition to mass-energy, linear momentum and orbital angular momentum. Poynting [2, p. 565] wrote:

“If we put *E* for the energy in unit volume and *G* for the torque per unit area, we have

(1.1) G=Eλ/2π”.

In formula (1.1), *G* is the density of the *distributed* torque, G [$J/m^{2}$]; that is, the internal angular momentum passing through a unit area per unit time, G [$J\times s/m^{2}\times s$]; that is, flux density of the internal angular momentum. E [$J/m^{3}$] is the volumetric density of the electromagnetic energy.

In the 20th century, this internal angular momentum of electromagnetic waves was discovered theoretically within the Lagrangian formalism and called spin. In contrast to the orbital angular momentum, which is the moment^2^ of the linear momentum **L** [J×s],

(1.2) $$L=r\times p,L^{ik}=x^{i}p^{k}-x^{k}p^{i}\;\left(i,j,k,\ldots \;\text{are}\;x,y,z\right)\text{,}$$

spin is a local quantity and is described by the spin tensor, which we denote by the letter upsilon. This spin tensor is given in many textbooks (e.g. [[3] (9.3.14)]):

(1.3) $$\Upsilon^{\lambda \mu \nu }=-A^{\lambda }F^{\mu \nu }+A^{\nu }F^{\lambda \nu },\left(\lambda ,\mu ,\alpha ,\ldots \;\text{are}\;t,x,y,z\right)\text{.}$$

Here $A^{\alpha }$ is the magnetic vector potential, and $F^{\lambda \nu }$ is the field-strength tensor. The sense of a spin tensor $\Upsilon^{\lambda \mu \nu }$ is as follows. The component $\Upsilon^{ijk}$ [$J\times s/m^{2}\times s$] is a flux density of spin in the direction of the $x^{k}$ axis. For example, $dS_{z}=dS^{xy}=\Upsilon^{xyz}da_{z}dt$ is the *z*-component of spin passing through the surface element $da_{z}$ in time dt. The component $\Upsilon^{ijt}$ [$J\times s/m^{3}$] is a volumetric density of spin. This means that $dS^{xy}=\Upsilon^{xyt}dV$ is the spin in the spatial element dV. The volumetric density $\Upsilon^{xyt}$ moves with velocity c along the z-axis. Thus $с\Upsilon^{xyt}=\Upsilon^{xyz}$.

As is known, the spin density $\Upsilon^{xyt}$ and energy density u satisfy the photon's ratio ℏ=ℏω/ω:

(1.4) $$\Upsilon^{xyt}=u/\omega \text{.}$$

It is remarkable that Poynting's formula (1.1) is just such a relation (1.4). Really, $G=\Upsilon^{xyz}=\Upsilon^{xyt}c$, E=u, and we have instead of (1.1) $\Upsilon^{xyt}c=u\lambda /2\pi$.

Weissenhoff [4] defined a *spin-fluid* as

“a fluid each element of which possesses besides energy and linear momentum also a certain amount of angular momentum, proportional – just as energy and the linear momentum – to the volume of the element”.

According to this definition, circularly polarized light is a spin-fluid.

Unfortunately, the spin tensor (1.3) was eliminated by the Belinfante-Rosenfeld procedure [5,6]. This procedure is described in detail in Refs. [[7], [8], [9], [10], [11]]. The spin tensor was excluded from the electromagnetic field theory. The spin tensor is absent from fundamental publications [[12], [13], [14]] devoted to the angular momentum of light. The spin tensor is absent from articles in specialized journals (e.g. Refs. [15,16]). Spin, discovered in the 19th century, is excluded from the classical theory of fields. Landau and Lifshitz write [17 § 16]

“We are considering classical (and not quantum) theory, and therefor do not include effects which are related to the spin”.

As a result, the total angular momentum of the electromagnetic field is defined as the orbital angular momentum (1.2), where dp=gdV, ***g*** [$N\times s/m^{3}$] is a linear momentum volumetric density, i.e. ***g = I*** $\left[\text{kg}/m^{2}\times s\right]$ is a Poynting vector. Simmonds & Guttmann [19, p. 222] wrote:

“If the electromagnetic field possesses a linear momentum density ***g***, the angular momentum density, computed in respect to the origin, would be r×g. The total angular momentum of the electromagnetic field is then

(1.5) $$J=\int r\times gdV=\int r\times \left(E\times H\right)dV/c^{2}”\text{.}$$

However, having excluded spin, physicists were faced with the need to exclude from the theory also plane waves, which are successfully used in studying the flows of energy and momentum (For example, Einstein used plane wave when considering the pressure of light on a mirror [[18] § 8]). The fact is that according to definition (1.5), there is no angular momentum in perfect circularly polarized plane electromagnetic waves. Simmonds & Guttmann [19, p. 223] write:

“For the plane wave **E** and **H** are mutually perpendicular and their cross product was parallel to the direction of propagation **k**. Thus, the linear momentum density for such waves is also in the direction of propagation. It follows that for such waves r×g must be perpendicular to the direction of propagation, and the total angular momentum of a plane wave can have no component of angular momentum parallel to the direction of propagation”.

To get rid of this unacceptable absence of angular momentum in plane waves due to definition (1.5), it was decided to abandon plane waves altogether. Simmonds & Guttmann [19, p. 223] write:

“One way out of this dilemma is to realize that perfect plane waves which fill all of space do not exist. Hence, we should not worry about that which cannot be observed”.

However physicists detected the angular momentum in a circularly polarized plane wave, which has a finite extent in the x and y directions. Heitler [20] wrote

“A plane wave travelling in *z*-direction and with infinite extension in the *xy*-directions can have no angular momentum about the *z*-axis, because E×H is in the z-direction and ${\left[r\times \left(E\times H\right)\right]}_{z}=0$. However, this is no longer the case for the wave with finite extension in the *xy*-plane. It can be shown that the wall of such a wave packet gives a finite contribution to the angular momentum about the *z*-axis”.

The point is the surface of such a circularly polarized light beam, no matter how large in diameter, contains longitudinal components of electromagnetic fields $E_{z},H_{z}$ due to the fact that the field lines are closed. These longitudinal components create an orbital electromagnetic momentum $p^{\varphi }$. In other words, a certain part of the electromagnetic mass rotates around the beam axis. The moment of the orbital momentum $J^{r\varphi }=rp^{\varphi }$ is directed along the beam axis. Simmonds and Guttmann [19, p.227] correctly describe this circulating mass flow in a circularly polarized wave contained within a finite cylinder around the z axis:

“The electric and magnetic fields can have a nonzero z-component only within the skin region of this wave. Having z-components within this region implies the possibility of a nonzero z-component of angular momentum within this region. Since the wave is identically zero outside the skin and constant inside the skin region, the skin region is the only one in which the z-component of angular momentum does not vanish”.

Thus, a circularly polarized beam has the angular momentum (1.5) J=∫r×IdV of the rotating mass. This angular momentum is directed along the beam axis $J_{z}=J^{r\varphi }$. However, we show that this angular momentum is *stationary*. The rotating mass has no velocity along the beam axis. Therefore, there is *no flux* of this angular momentum. So, the angular momentum flux density that Poynting designated *G* absents in the beam, according to definition (1.5). Therefore, using a beam instead of a plane wave does not help to explain the existence of angular momentum flux in electromagnetic radiation based on definition (1.5).

## 2 Calculation of the energy-momentum tensor of the beam

Jackson considers a circularly polarized light beam [21, p. 350]:

“If the amplitude modulation is slowly varying (the wave is many wavelengths board), the electric and magnetic fields are given approximately by

(2.1) $$E\left(x,y,z\right)≃\left[\left(e_{x}+ie_{y}\right)+\left(e_{z}/k\right)\left(i\partial_{x}-\partial_{y}\right)\right]E_{0}\left(x,y\right)e^{ikz-i\omega t},B≃-iE/c\text{,}$$

where $e_{x},e_{y},e_{z}$ are unit vectors in the *x, y, z* directions”.

The expression (2,1) means that the components of the electromagnetic field-strength tensor $F_{\alpha \beta }$ of the beam are

(2.2) $$\left\{F_{tx}=E_{0},F_{ty}=iE_{0},F_{tz}=\frac{\left(i\partial_{x}-\partial_{y}\right)E_{0}}{k},F_{zy}=-\frac{iE_{0}}{c},F_{xz}=\frac{E_{0}}{c},F_{yx}=\frac{\left(\partial_{x}+i\partial_{y}\right)E_{0}}{ck}\right\}e^{ikz-i\omega t}\text{.}$$

Using cylindrical coordinates r,φ,z gives (for $E_{0}\left(r\right)$):

(2.3) $$\left\{F_{tr}=E_{0},F_{t\varphi }=irE_{0},F_{tz}=\frac{i\partial_{r}E_{0}}{k},F_{z\varphi }=-\frac{irE_{0}}{c},F_{rz}=\frac{E_{0}}{c},F_{\varphi r}=\frac{r\partial_{r}E_{0}}{ck}\right\}e^{ikz-i\omega t+i\varphi }\text{.}$$

The transition from the tensor $F_{\alpha \beta }$ to the tensor density $F_{\wedge }^{\alpha \beta }$ is carried out by the *conjugation* [22,23], which involves raising the coordinate indices and includes the factor ${\sqrt{g}}_{\wedge }=rc$ where g is the determinant of the metric tensor of the coordinates used, and includes the factor $\sqrt{\varepsilon_{0}/\mu_{0}}$, which provides the common change of units. (We avoid the use of Gothic fonts adopted to denote densities by using the subscript ∧) As a result of the conjugation, the components $F_{\wedge }^{\alpha \beta }$ are

(2.4) $$\left\{F_{\wedge }^{rt}=r\varepsilon_{0}E_{0},F_{\wedge }^{\varphi t}=i\varepsilon_{0}E_{0},F_{\wedge }^{zt}=\frac{ir\varepsilon_{0}\partial_{r}E_{0}}{k},F_{\wedge }^{z\varphi }=-iE_{0}c\varepsilon_{0},F_{\wedge }^{rz}=rE_{0}c\varepsilon_{0},F_{\wedge }^{\varphi r}=\frac{\partial_{r}E_{0}c\varepsilon_{0}}{k}\right\}e^{ikz-i\omega t+i\varphi }$$

The energy-momentum tensor [17,21] is

(2.5) $$T_{\wedge }^{\lambda \mu }=-g^{\lambda \alpha }F_{\alpha \beta }F_{\wedge }^{\mu \beta }+g^{\lambda \mu }F_{\alpha \beta }F_{\wedge }^{\alpha \beta }/4\text{.}$$

However, for the Jackson's beam (2.1)

(2.6) $$\begin{array}{l} \;<F_{\alpha \beta }F_{\wedge }^{\alpha \beta }>=R\left\{{\bar{F}}_{rt}F_{\wedge }^{rt}+{\bar{F}}_{\varphi t}F_{\wedge }^{\varphi t}+{\bar{F}}_{zt}F_{\wedge }^{zt}+{\bar{F}}_{z\varphi }F_{\wedge }^{z\varphi }+{\bar{F}}_{rz}F_{\wedge }^{rz}+{\bar{F}}_{\varphi r}F_{\wedge }^{\varphi r}\right\}\\ \;=R\left\{-r\varepsilon_{0}E_{0}^{2}-r\varepsilon_{0}E_{0}^{2}-r\varepsilon_{0}{\left(\partial_{r}E_{0}\right)}^{2}/k^{2}+r\varepsilon_{0}E_{0}^{2}+r\varepsilon_{0}E_{0}^{2}+r\varepsilon_{0}{\left(\partial_{r}E_{0}\right)}^{2}/k^{2}\right\}=0 \end{array}$$

So the mass-energy volumetric density represented by $T_{\wedge }^{tt}$ is

(2.7) $$T_{\wedge }^{tt}=-g^{tt}R\left\{{\bar{F}}_{tr}F^{tr}+{\bar{F}}_{t\varphi }F^{t\varphi }+{\bar{F}}_{tz}F^{tz}\right\}/2=\left({2r\varepsilon_{0}E_{0}^{2}+r\varepsilon_{0}\left(\partial_{r}E_{0}\right)}^{2}/k^{2}\right)/2c^{2}\text{.}$$

Components of the Poynting vector are $I_{\wedge }^{z}=T_{\wedge }^{tz}=-g^{tt}F_{t\beta }F_{\wedge }^{z\beta }$, $I_{\wedge }^{\varphi }=T_{\wedge }^{t\varphi }=-g^{tt}F_{t\beta }F_{\wedge }^{\varphi \beta }$. So

(2.8) $$I_{\wedge }^{z}=-g^{tt}R\left\{{\bar{F}}_{tr}F_{\wedge }^{zr}+{\bar{F}}_{t\varphi }F_{\wedge }^{z\varphi }\right\}/2=-\left\{E_{0}\left(-rE_{0}c\varepsilon_{0}\right)-irE_{0}\left(-iE_{0}c\varepsilon_{0}\right)\right\}/2c^{2}=r\varepsilon_{0}E_{0}^{2}/c\text{,}$$

(2.9) $$I_{\wedge }^{\varphi }=-g^{tt}R\left\{{\bar{F}}_{tr}F_{\wedge }^{\varphi r}+{\bar{F}}_{tz}F_{\wedge }^{\varphi z}\right\}/2=-\left\{E_{0}\frac{\partial_{r}E_{0}c\varepsilon_{0}}{k}-\frac{i\partial_{r}E_{0}}{k}iE_{0}c\varepsilon_{0}\right\}/2c^{2}=-\varepsilon_{0}\partial_{r}E_{0}^{2}/2\omega \text{.}$$

## 3 Volumetric densities and flux densities

Component (2.7) $T_{\wedge }^{tt}$ $\left[\text{kg}/m^{2}\times \text{radian}\right]$ is the volumetric density of the mass in a cylindrical coordinate system. It shows that the mass consists of two parts:

(3.1) $$T_{\wedge }^{tt}={\underset{1}{T}}_{\wedge }^{tt}+{\underset{2}{T}}_{\wedge }^{tt},{\underset{1}{T}}_{\wedge }^{tt}=r\varepsilon_{0}E_{0}^{2}\left(r\right)/c^{2},{\underset{2}{T}}_{\wedge }^{tt}=r\varepsilon_{0}{\left(\partial_{r}E_{0}\right)}^{2}/\left(2c^{2}k^{2}\right)\text{.}$$

The main part of the mass is represented by the first term ${\underset{1}{T}}_{\wedge }^{tt}$. This part of the mass fills the entire volume of the beam. The second small part of the mass, proportional to $1/k^{2}$, is represented by the second term ${\underset{2}{T}}_{\wedge }^{tt}$. It is located within the skin region, where the derivative $\partial_{r}E_{0}$ exists.

The main part of the mass moves along the beam axis at the speed of light $v^{z}=c$ and provides the mass flux density in the beam in the direction of the axis. This mass flux density is represented by the Poynting vector (2.8) $I_{\wedge }^{z}=T_{\wedge }^{tz}=r\varepsilon_{0}E_{0}^{2}/c$ [kg/m×radian×s]. It can be obtained by multiplying the volumetric density of the main part of mass by the speed of light: $I_{\wedge }^{z}={\underset{1}{T}}_{\wedge }^{tt}c$. The second part of the beam mass, represented by the second term ${\underset{2}{T}}_{\wedge }^{tt}$, does not participate in the motion along the axis. This is proved by the fact that the expression for the mass flux density along the axis, $I_{\wedge }^{z}$, contains the first part of the mass and only it.

The second part of the mass, located in the skin region, moves in the direction of the angular coordinate. This is proved by the fact that the mass flux density in the direction of the angular coordinate, i.e. the component $I_{\wedge }^{\varphi }$ of the Poynting vector, is determined by the derivative $\partial_{r}E_{0}$: $I_{\wedge }^{\varphi }=T_{\wedge }^{t\varphi }=-\varepsilon_{0}\partial_{r}E_{0}^{2}/2\omega$ $\left[\text{kg}/m^{2}\times s\right]$. This means that the second part of the mass creates a flow of mass that rotates around an axis. $I_{\wedge }^{\varphi }$ is the volumetric density of the momentum in the flow. The volumetric density of the angular momentum is $j_{\wedge }^{r\varphi }=rI_{\wedge }^{\varphi }$. The total angular momentum of this azimuthal mass flow can be calculated: $J^{r\varphi }=\int j_{\wedge }^{r\varphi }dV^{\wedge }$. This angular momentum, as a vector, is directed along the z-axis. However, this angular momentum does not move along the z-axis. The velocity of the second part of the mass in the z-direction is zero, $v^{z}=0$. The angular momentum flux density in z-direction equals zero: $j_{\wedge }^{r\varphi z}=j_{\wedge }^{r\varphi }v^{r}=0$. So the angular momentum flux equals zero: $\int j_{\wedge }^{r\varphi z}da_{z}^{\wedge }=0$. Therefore, there is no flow of the angular momentum $J^{r\varphi }$ along the beam axis. This is the main our result.

## 4 Spin tensor

It is natural to recognize that the flow of the angular momentum discovered by the classics in 19th century is contained in the main mass of the beam. But the Maxwell's electrodynamics does not catch sight of this angular momentum as well as of angular momentum in a circularly polarized plane wave. So we are forced to recognize that the flow of the angular momentum is the flow of spin.

To calculate spin flux density (1.3), $\Upsilon_{\wedge }^{r\varphi z}=-A^{r}F_{\wedge }^{\varphi z}+A^{\varphi }F_{\wedge }^{rz}$, we use the vector potentials:

(4.1) $$A_{r}=\int F_{tr}dt=iE_{0}e^{ikz-i\omega t+i\varphi }/\omega ,A_{\varphi }=\int F_{\varphi r}dt=-rE_{0}e^{ikz-i\omega t+i\varphi }/\omega \text{.}$$

Raising the indices gives:

(4.2) $$A^{r}=g^{rr}A_{r}=-iE_{0}e^{ikz-i\omega t+i\varphi }/\omega ,A^{\varphi }=g^{\varphi \varphi }A_{\varphi }=E_{0}e^{ikz-i\omega t+i\varphi }/r\omega \text{.}$$

So the spin flux density is

(4.3) $${ϒ}_{\wedge }^{r\varphi z}=R\left\{-{\bar{A}}^{r}F_{\wedge }^{\varphi z}+{\bar{A}}^{\varphi }F_{\wedge }^{rz}\right\}/2=\left\{\left(-iE_{0}/\omega \right)iE_{0}c\varepsilon_{0}+\left(E_{0}/r\omega \right)rE_{0}c\varepsilon_{0}\right\}/2=E_{0}^{2}c\varepsilon_{0}/\omega \text{.}$$

To integrating, it is need find the physical component

(4.4) $${ϒ}_{\wedge }^{r\hat{\varphi }z}={ϒ}_{\wedge }^{r\varphi z}\sqrt{g_{\varphi \varphi }}={ϒ}_{\wedge }^{r\varphi z}r=rE_{0}^{2}c\varepsilon_{0}/\omega \text{.}$$

Integrating of the spin flux density gives the spin flux:

(4.5) $$dJ^{r\hat{\varphi }}/dt=\int {ϒ}_{\wedge }^{r\hat{\varphi }z}da_{z}^{\wedge }=\int_{0}^{R}\int_{0}^{2\pi }{ϒ}_{\wedge }^{r\hat{\varphi }z}drd\varphi =\int_{0}^{R}2\pi rE_{0}^{2}c\varepsilon_{0}dr/\omega =\pi R^{2}\varepsilon_{0}E_{0}c/\omega \;\left[J\right]$$

The electromagnetic power of the circularly polarized beam is

(4.6) $$dW/dt=\pi R^{2}\varepsilon_{0}E_{0}c\;\left[W\right]\text{.}$$

So, $J^{r\hat{\varphi }}=W/\omega$ just as ℏ=ℏω/ω.

## 5 The use of the spin tensor

The absence of angular momentum flux in a beam with a plane phase front, according to Maxwell's electrodynamics, makes Beth's famous experiment [24] with a half-wave plate inexplicable. Passing through a half-wave plate, light changes its helicity. Accordingly, the direction of the spin changes to the opposite direction, and the twice amount of spin angular momentum is transferred to the plate. This is registered as torque acting on the plate. The use of the spin tensor explains Beth's result [[25], [26], [27], [28], [29]].

The spin tensor also made it possible to explain other classical experiments with a half-wave plate, which were inexplicable by Maxwell's electrodynamics [[30], [31], [32], [33]]. In these experiments, the half-wave plate rotates in its plane, and the torque transmitted to it does work. This changes the frequency of light as it passes through the rotating plate. The change in frequency is recorded using interference.

The spin tensor made it possible to detect previously unknown spin radiation in the well-studied field of a rotating electric dipole [[34], [35], [36], [37], [38]]. This spin radiation is directed predominantly along the dipole rotation axis, in the direction along which the radiation is circularly polarized.

It is important that, according to Poynting's formula (1.1), the spin density is locally proportional to the energy density. Formula (1.1) is well known and not disputed. Article [2] is included in book [12]. Also Crawford [39] emphasizes: “A traveling plane wave can transfer not only energy and linear momentum, but also angular momentum”. Accordingly, the spin tensor is widely used to study spin fluxes in plane waves. Four articles [[40], [41], [42], [43]] consider the reflection and/or absorption of spin during the interaction of light with a moving mirror or absorber under normal light incidence. A precise balance has been demonstrated for spin, energy, and momentum in incident, reflected and absorbed waves when the spin tensor is used in parallel with the energy-momentum tensor (2.5). This takes into account the accumulation of spin in space, which is vacated by a moving surface and filled with radiation. The motion of the absorber surface requires the application of the Lorentz transformation to the spin tensor and energy-momentum tensor [40].

Spin is not transferred to an ideal mirror when light is incident at normal angles. However, when light is incident and is reflected at an angle, the direction of light changes. At the same time, the direction of the spins of the photons of this light changes, since the spin of a photon is always parallel to the momentum of the photon. (A photon differs from a ball, whose angular momentum does not change when it bounces off a mirror.) Therefore, the mirror receives an angular momentum directed parallel to the surface of the mirror when circularly polarized light is incident at an angle. If the mirror rotates so that the angular velocity is directed parallel to the mirror, work is done that changes the frequency of the light in the same way as it occurs when a half-wave plate rotates [[30], [31], [32], [33]]. In Ref. [44], the reflection of light from a rotating mirror cylinder is considered in an experiment that is a modification of Lloyd's experiment.

In the article [45] a rotating absorber of circularly polarized light is considered. It is shown that the heating of a rotating absorber differs from the heating of a stationary absorber by exactly the amount of energy that corresponds to the work of the spin during its absorption.

Spin absorption induces an unusual mechanical stress in the absorber, described by an antisymmetric stress tensor [46].

## 6 Conclusion

The angular momentum of light, discovered in the 19th century, is spin of light, and it is not described by the Maxwell's electrodynamics, which ignores the spin tensor. The spin tensor should be included in the theory of electromagnetism along with the energy-momentum tensor. The use of the spin tensor has generated numerous results that go beyond the boundaries of the Maxwell's electrodynamics.

The topic is of interest in practical issues in optical micromanipulation and of theoretical interest in the foundations of field theory and classical electrodynamics.

The original idea for this article was bravely published by Robert Romer [47].

## Ethical Approval

Not applicable.

## Funding

Not applicable.

## Availability of data and materials

Not applicable.

## CRediT authorship contribution statement

**R.I. Khrapko:** Conceptualization.

## Declaration of competing interest

The authors declare that they have no known competing financial interests or personal relationships that could have appeared to influence the work reported in this paper.

## Footnotes

2 For the term ‘moment’ see, for example, the Penguin Dictionary of Physics.
